# Pigmented Skin Disorders in High‐Altitude: Cross‐Sectional Epidemiological Analysis in the General Population of Lhasa, China

**DOI:** 10.1111/jocd.70030

**Published:** 2025-02-08

**Authors:** Mingming Xu, Zha Zhen, Ying Chen, Shuo Zhang, Jing Li, Nan Li, Ruiyu Li, Suolang Quzong, Liming Huang, Wei Zhang

**Affiliations:** ^1^ Hospital for Skin Diseases Institute of Dermatology, Chinese Academy of Medical Science and Peking Union Medical College Nanjing Jiangsu China; ^2^ People’s Hospital of Xizang Autonomous Region Lhasa China; ^3^ Epidemiology and Biostatistics Department, School of Public Health Kunming Medical University Kunming Yunnan China; ^4^ Department of Dermatology First Affiliated Hospital of Kunming Medical University Kunming Yunnan China

**Keywords:** cross‐sectional study, high‐altitude area, melasma, plateau facial telangiectasia, vitiligo

## Abstract

**Background:**

Previous studies showed the prevalence of melasma, plateau facial telangiectasia, and vitiligo varied in different regions but were absent in high‐altitude areas.

**Aims:**

To investigate the prevalence of three common pigmented skin disorders (melasma, plateau facial telangiectasia, and vitiligo) among the general population in Lhasa, aiming to provide a scientific basis for their prevention and management in high‐altitude areas.

**Methods:**

From May 2021 to October 2021, multistage stratified cluster random sampling was conducted in Lhasa to carry out a questionnaire and a second on‐site physical examination, and the results were statistically analyzed.

**Results:**

The study included 4988 participants, revealing a prevalence of 14.94% for melasma, 17.14% for plateau facial telangiectasia, and 0.38% for vitiligo. Notably, the prevalence of melasma and plateau facial telangiectasia were significantly higher among women, particularly those aged 31–50 years, compared to men. Urban residents also showed a higher prevalence than their rural counterparts.

**Conclusions:**

Our study concludes that the prevalence of the three pigmented skin disorders in Lhasa is notably higher than in lower‐altitude areas, with UV radiation being a significant risk factor. Our findings underscore the need for enhanced public health interventions, including screening, education, and prevention efforts, to mitigate the impact of these skin disorders in high‐altitude regions. Our research contributes valuable insights toward the understanding and management of pigmented skin disorders in such unique environments. It may also provide an easy‐to‐use epidemiological survey method for socioeconomically underdeveloped areas.

## 1 Introduction

The global prevalence of pigmented skin disorders varies significantly. Studies have demonstrated that the prevalence of melasma ranges from 5% to 46% [1, 2, 3] depending on the population examined. A population‐based study involving 1400 residents revealed a 10% prevalence of plateau facial telangiectasia [4]. Meanwhile, the worldwide prevalence of vitiligo is estimated to range from 0.4% to 2% [5], with a reported prevalence of 0.56% in China [6]. The variation in the prevalence of pigmented skin disorders across different regions may be attributed to factors such as ethnicity, skin type, and environmental conditions [7]. Ultraviolet (UV) radiation serves as a primary trigger for these conditions. Lhasa, situated in the central part of the Xizang Plateau with 867 891 permanent residents, stands as one of the highest cities in the world at an elevation of 3650 m above sea level and experiences intense UV radiation exposure. Consequently, photodamage associated with pigmented disorders is exceedingly common, presenting a significantly elevated risk compared to lower‐altitude areas. Despite this, research on the prevalence of pigmented disorders in high‐altitude areas remains scarce. Thus, an epidemiological study focusing on three common pigmented skin disorders was conducted in Lhasa to explore their prevalence among the general population, aiming to furnish a scientific foundation for their prevention in high‐altitude areas.

## 2 Methods

### 2.1 Study Design and Participants

In this population‐based, cross‐sectional study, we estimated the prevalence of three common pigmented skin disorders among the general population in Lhasa, using a multistage stratified cluster random sampling methodology. In the initial stage, one district and one county (Chengguan District and Linzhou County) were randomly chosen from the eight districts/counties in Lhasa. Subsequently, in the second stage, four townships (Bianjiaolin Township, Pangduo Township, Najin Township, and Zhaxi Township) were randomly selected from the 22 streets/townships within Chengguan District and Linzhou County. In the final stage, residents from four communities (Xiongga Community, Tuanjiexincun Community, Gaba Community, and Zangre Community) and four villages (Dangjie Village, Sekang Village, Ningbu Village, Jiage Village) were randomly chosen from among 18 communities/villages in the selected townships. During the field investigation, both paper and electronic questionnaires were utilized. The sample size calculation was based on the following formula:

$$n={\left[\frac{57.3\mu_{\alpha /2}}{\sin^{-1}\left[\delta /\sqrt{\pi \left(1-\pi \right)}\right]}\right]}^{2}$$

**FIGURE 1:**
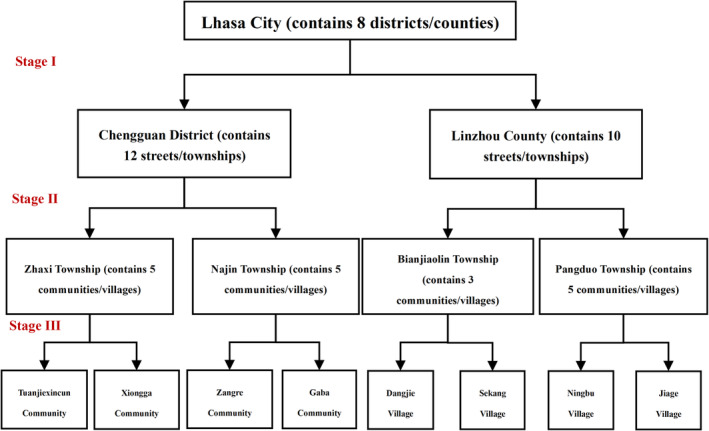
Multistage stratified cluster random sampling procedure.

**FIGURE 2:**
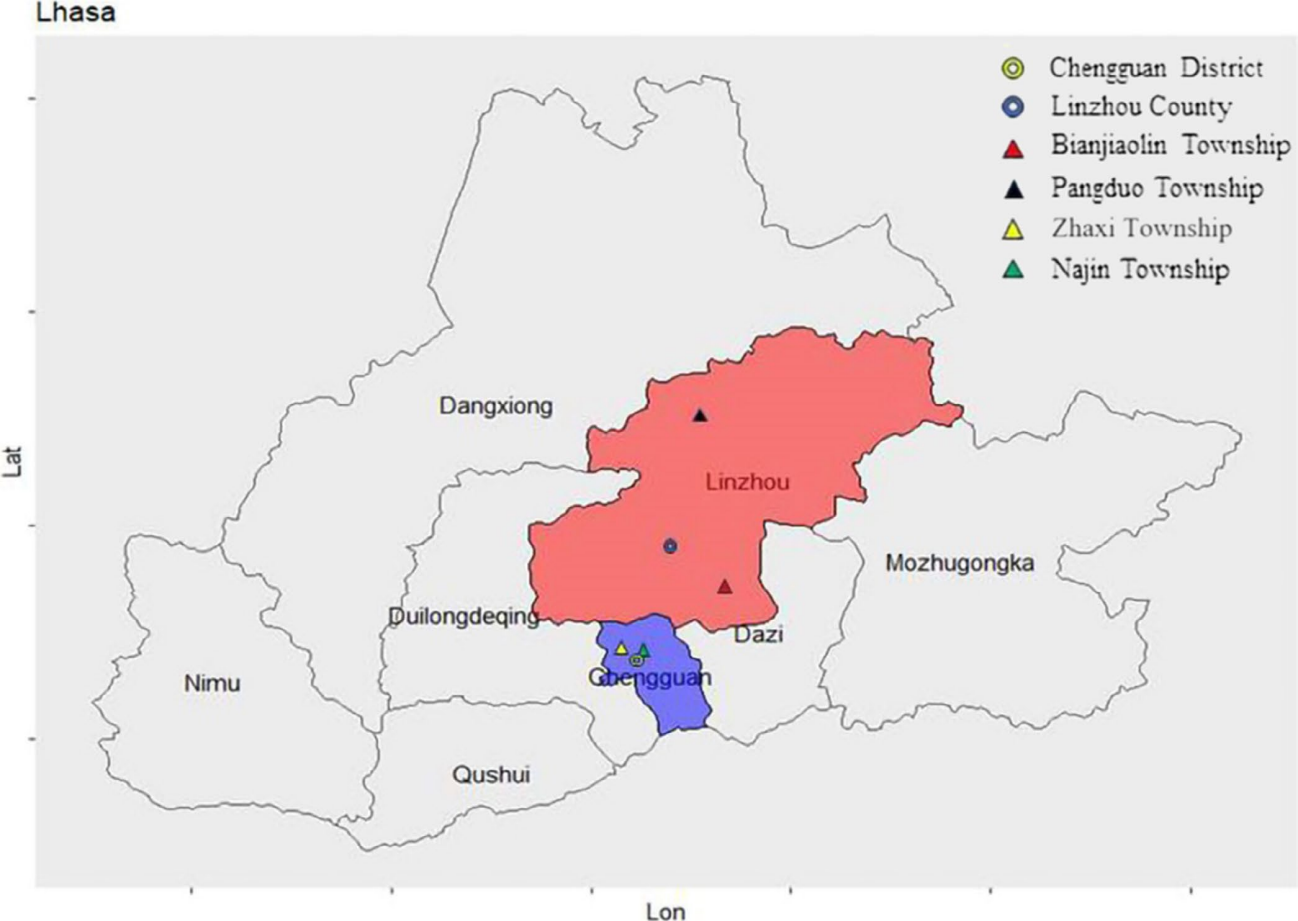
Geographical distribution of the investigated areas in Lhasa.

*π* represented the prevalence of vitiligo in US [8] (0.16%). Considering a permissible relative error (ε) of 10% and a permissible absolute error *δ* = *ε* × *p* = 0.1 × 0.0016, the total sample size *N* ≈ 5000. Participants in our study were given the choice to either complete the questionnaires independently or with assistance from an investigator. A total of 5234 individuals were invited to participate in the study. After excluding incomplete or duplicate questionnaires, the analysis ultimately included 4988 participants. The questionnaire achieved a response rate of 95.30% (Figures 1 and 2).

The study was approved by Ethics Committee of the Xizang Autonomous Region People's Hospital, and informed consent was obtained with patient signatures.

### 2.2 Data Collection and Definitions

During the preparatory stage, to enhance the quality of our study, we involved staff members from four villages and four communities in the on‐site training for investigators. This allowed us to timely address issues encountered during the preliminary investigation and refine our investigative approach.

The fieldwork phase spanned from May to October 2021. Investigators visited villages and communities to support the survey process, conducting on‐site questionnaires, collecting completed questionnaires, and performing initial screenings for pigmented skin disorders. Subsequently, dermatologists provided assistance in finalizing the survey and rectifying any errors identified in the field.

**FIGURE 3:**
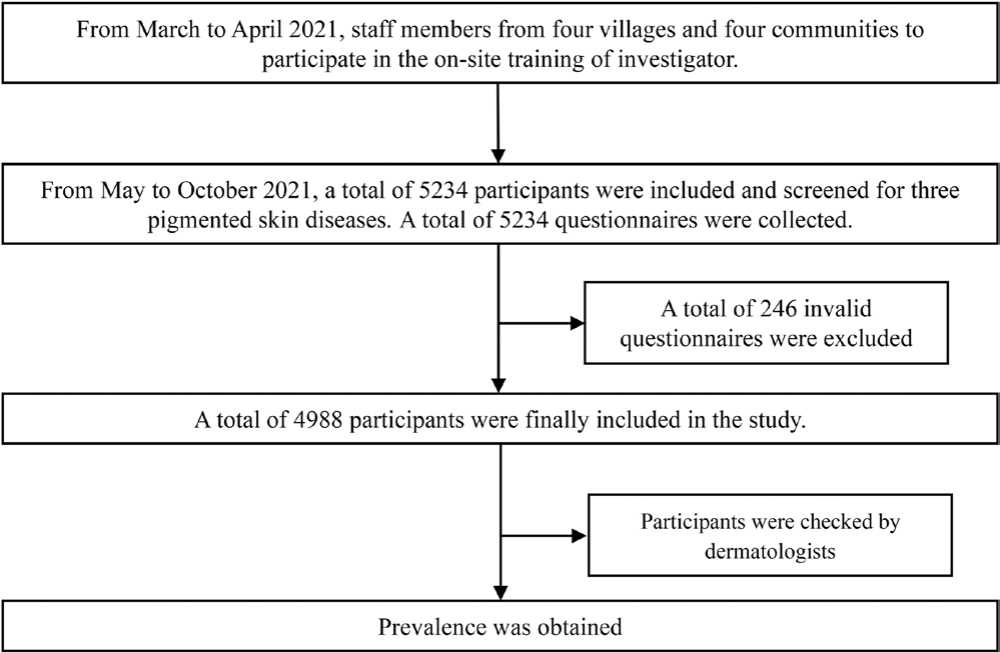
Study flowchart for disposition of the patients.

Upon collection and organization of the questionnaires, investigators reviewed them, and invalid questionnaires were excluded (Figure 3).

### 2.3 Statistical Analysis

All analyses were performed using IBM SPSS Statistics version 20.0 software (IBM Corp., Armonk, NY, USA). Descriptive statistics were calculated for participant demographic and clinical characteristics and reported overall and for the participants. We estimated the prevalence of three common pigmented skin disorders among all participants. Continuous variables were presented as the mean ± standard deviation (SD), and categorical variables were presented as counts and percentages (%). Comparisons between the participants were assessed using the *t*‐test for continuous variables. The significance level was 2‐sided *p* < 0.05 and was presented for descriptive purposes.

## 3 Results

A total of 5234 questionnaires were distributed. After a series of screening and confirmation, and exclusion of invalid questionnaires, 4988 participants were included in the final analysis, with an overall response rate of 95.30%, including 2761 women (55.4%) and 2227 men (44.6%). There were 1229 (24.6%), 855 (17.14%), and 19 (0.38%) patients initially diagnosed with melasma, plateau facial telangiectasia and vitiligo, respectively. There were 755 (14.94%), 855 (17.14%) and 19 (0.38%) patients finally diagnosed with melasma, plateau facial telangiectasia, and vitiligo in the second physical examination, respectively. In this study, the sensitivity of melasma screening was 99.8%, the specificity was 88.9%, the Kappa value was 0.698 (*p* < 0.001), and the AUC was 0.942 (*p* < 0.001), and the sensitivity and specificity of screening for plateau facial telangiectasia and vitiligo were both 100%, with the Kappa value was 1.000 (*p* < 0.001) and the AUC was 1.000 (*p* < 0.001).

The average age of the participants was 39.48 ± 16.70 years old. The 4373 Zang accounted for the highest proportion among all ethnic groups (87.7%), the Han was 550 (11.0%), and other ethnic groups were 64 (1.3%). The urban population was 2111 (42.3%), and the rural population was 2877 (57.7%). There were 2650 farmers and herdsmen (53.13%), 871 other employees (17.46%), 575 workers or self‐employed people or drivers (11.53%), 477 students (9.56%), and 415 police or medical staff or civil servants (8.32%).

The prevalence of melasma in this study was 14.94% (Table 1). The prevalence in women (20.4%) was significantly higher than that in men (8.2%), especially among women aged 31 to 40 years old (28.4%, 95% CI 24.9%–32.0%). Before the age of 31–40 years, the prevalence increased with age and then decreased with age. There were statistically significant differences in the prevalence of melasma among different age groups, ethnic groups, occupations, and regions (*p* < 0.05). There were statistical differences in the prevalence of melasma among women of different ethnic groups (*p* < 0.05), Zang women (18.8%, 95% CI 17.3%–20.4%) had the lowest prevalence of melasma, while Han (34.6%, 95% CI 28.5%–40.6%) women had a higher prevalence than Zang women, and the highest prevalence was found in the other ethnic groups (35.5%, 95% CI 17.6%–53.3%). Police, medical staff, and civil servants had the highest prevalence among all occupations (17.9% for men and 42.9% for women), and students had the lowest prevalence (0.9% for men and 4.9% for women). The prevalence of melasma among urban residents was higher than that areas among rural residents (14.1% for men and 38.1% for women).

**TABLE 1:** Prevalence of melasma.

Category	Male				Female				*χ* ^2^	*p*
	*n*	Prevalence	95% CI		*n*	Prevalence	95% CI			
Age groups										
< 18	0	0	0.0	0.0	0	0.0	0.0	0.0	152.458	< 0.001
18–25	14	5.9	2.9	8.9	58	21.2	16.3	26.0		
26–30	28	12.0	7.8	16.2	87	28.1	23.0	33.1		
31–40	50	9.2	6.7	11.6	178	28.4	24.9	32.0		
41–50	34	8.0	5.4	10.6	153	25.9	22.4	29.5		
51–60	37	11.8	8.2	15.4	60	13.8	10.5	17.0		
> 60	19	9.8	5.6	14.1	27	10.7	6.8	14.5		
Total	182	8.2	7.0	9.3	563	20.4	18.9	21.9		
*p*	< 0.001				< 0.001					
Ethnic group										
Zang	143	7.6	6.4	8.8	468	18.8	17.3	20.4	26.296	< 0.001
Han	35	11.4	7.8	15.0	84	34.6	28.5	40.6		
Other	4	12.1	4.0	23.9	11	35.5	17.6	53.3		
Total	182	8.2	7.0	9.3	563	20.4	18.9	21.9		
*p*	0.054				< 0.001					
Occupation										
Farmer/Herdsman	61	5.3	4.0	6.5	165	11.1	9.5	12.7	398.556	< 0.001
Worker/Self‐employed/driver	42	11.4	8.1	14.6	69	33.5	27.0	40.0		
Police/Medical Staff/Civil servant	33	17.9	12.3	23.5	99	42.9	36.4	49.3		
Student	2	0.9	0.3	2.1	12	4.9	2.2	7.7		
Other	44	15.7	11.4	19.9	218	36.9	33.0	40.9		
Total	182	8.2	7.0	9.3	563	20.4	18.9	21.9		
*p*	< 0.001				< 0.001					
Region										
Urban	130	14.1	11.8	16.3	453	38.1	35.4	40.9	463.268	< 0.001
Rural	52	4.0	2.9	5.1	110	7.0	5.7	8.3		
Total	182	8.2	7.0	9.3	563	20.4	18.9	21.9		
*p*	< 0.001				< 0.001					

Abbreviation: 95% CI = 95% confidence interval.

The prevalence of plateau facial telangiectasia in this study was 17.14% (Table 2). The prevalence in women (21.2%) was much higher than that in men (12.1%). There was a statistical difference between women's age groups in the prevalence of plateau facial telangiectasia (*p* < 0.05). The prevalence was highest among women aged 41–50 years (26.3%, 95% CI 22.7%–29.8%). There were statistically significant differences in the prevalence of plateau facial telangiectasia among different age groups, ethnic groups, occupations, and regions (*p* < 0.05). Among men, the highest prevalence was among Han people (16.6%, 95% CI 12.4%–20.8%) and police, medical staff, and civil servants (16.6%, 95% CI 12.4%–20.8%). Among women, other ethnic groups had the highest prevalence (51.6%, 95% CI 33.0%–70.2%), and other employees had the highest prevalence (34.6%, 95% CI 30.7%–38.4%). Zang men and women have the lowest prevalence (11.3% for men and 19.5% for women), and the prevalence among urban residents was higher than that among village residents.

**TABLE 2:** Prevalence of plateau facial telangiectasia.

Category	Male				Female				*χ* ^2^	*p*
	*n*	Prevalence	95% CI		*n*	Prevalence	95% CI			
Age groups										
< 18	28	10.0	6.5	13.5	29	10.7	7.0	14.4	44.255	< 0.001
18–25	29	12.2	8.0	16.4	64	23.4	18.3	28.4		
26–30	41	17.6	12.7	22.5	78	25.2	20.3	30.0		
31–40	61	11.2	8.5	13.8	154	24.6	21.2	28.0		
41–50	44	10.4	7.5	13.3	155	26.3	22.7	29.8		
51–60	42	13.4	9.6	17.2	80	18.3	14.7	22.0		
> 60	24	12.4	7.7	17.1	26	10.3	6.5	14.0		
Total	269	12.1	10.7	13.4	586	21.2	19.7	22.8		
*p*	0.130				0.000					
Ethnic group										
Zang	214	11.3	9.9	12.8	485	19.5	17.9	21.1	35.402	< 0.001
Han	51	16.6	12.4	20.8	85	35.0	28.9	41.0		
Other	4	12.1	0.4	23.9	16	51.6	33.0	70.2		
Total	269	12.1	10.7	13.4	586	21.2	19.7	22.8		
*p*	0.041				0.000					
Occupation										
Farmer/Herdsman	117	10.1	8.4	11.8	207	13.9	12.1	15.7	168.683	< 0.001
Worker/Self‐employed/driver	50	13.6	10.0	17.1	71	34.5	27.9	41.0		
Police/Medical staff/Civil servant	31	16.8	11.4	22.3	79	34.2	28.0	40.4		
Student	27	11.6	7.4	15.7	25	10.2	6.4	14.1		
Other	44	15.7	11.4	19.9	204	34.6	30.7	38.4		
Total	269	12.1	10.7	13.4	586	21.2	19.7	22.8		
*p*	0.014				0.000					
Region										
Urban	140	15.2	12.8	17.5	412	34.7	32.0	37.4	209.081	< 0.001
Rural	129	9.9	8.3	11.5	174	11.1	9.5	12.6		
Total	269	12.1	10.7	13.4	586	21.2	19.7	22.8		
*p*	0.000				0.000					

Abbreviation: 95% CI = 95% confidence interval.

The prevalence of vitiligo in this study was 0.38% (Table 3). The prevalence of vitiligo in women (0.5%) was slightly higher than that in men (0.2%). The prevalence of vitiligo was not significantly different among different age groups both in men and women (*p* > 0.05). There was an age difference in the prevalence (*p* < 0.05), with the highest prevalence in males aged under 18 years (1.1%, 95% CI 0.1%–2.3%). There was no significant correlation between ethnic groups, occupations, and regions in the prevalence of vitiligo (*p* > 0.05).

**TABLE 3:** Prevalence of vitiligo.

Category	Male				Female				*χ* ^2^	*p*
	*n*	Prevalence	95% CI		*n*	Prevalence	95% CI			
Age groups										
< 18	3	1.1	0.1	2.3	1	0.4	0.1	1.1	10.409	0.106
18–25	0	0.0	0.0	0.0	1	0.4	0.1	1.1		
26–30	0	0.0	0.0	0.0	2	0.6	0.3	1.5		
31–40	1	0.2	0.1	0.5	7	1.1	0.3	1.9		
41–50	1	0.2	0.1	0.7	2	0.3	0.1	0.8		
51–60	0	0.0	0.0	0.0	0	0.0	0.0	0.0		
> 60	0	0.0	0.0	0.0	1	0.4	0.1	1.2		
Total	5	0.2	0.1	0.4	14	0.5	0.2	0.8		
*p*	0.041				0.462					
Ethnic group										
Zang	4	0.2	0.0	0.4	13	0.5	0.2	0.8	0.500	0.779
Han	1	0.3	0.1	1.0	1	0.4	0.1	1.2		
Other	0	0.0	0.0	0.0	0	0.0	0.0	0.0		
Total	5	0.2	0.1	0.4	14	0.5	0.2	0.8		
*p*	0.935				0.625					
Occupation										
Farmer/Herdsman	2	0.2	0.1	0.4	5	0.3	0.1	0.6	6.679	0.154
Worker/Self‐employed /driver	1	0.3	0.1	0.8	0	0.0	0.0	0.0		
Police/Medical staff/Civil servant	0	0.0	0.0	0.0	4	1.7	0.1	3.4		
Student	1	0.4	0.1	1.3	0	0.0	0.0	0.0		
Other	1	0.4	0.1	1.1	5	0.8	0.1	1.6		
Total	5	0.2	0.1	0.4	14	0.5	0.2	0.8		
*p*	0.490				0.150					
Region										
Urban	1	0.1	0.0	0.3	8	0.7	0.2	1.1	0.199	0.656
Rural	4	0.3	0.1	0.6	6	0.4	0.1	0.7		
Total	5	0.2	0.1	0.4	14	0.5	0.2	0.8		
*p*	0.411				0.285					

Abbreviation: 95% CI = 95% confidence interval.

## 4 Discussion

Melasma frequently appears on the cheeks, chin, forehead, and other symmetrical parts of the body [9, 10]. Telangiectasia, which are superficial skin vessels visible to the naked eye, typically manifests as red linear and arborizing formations, with the nasal alae, nose, and mid‐cheeks being the most common areas of occurrence. Vitiligo is primarily characterized by milky white macules with distinct edges and patchy depigmentation of the skin and/or hair, resulting from the loss of epidermal melanocytes [11, 12, 13]. These conditions significantly impact the physical and psychological well‐being of patients, profoundly diminishing their quality of life [14, 15, 16].

Our research provides critical insights into the prevalence of three common pigmented skin disorders in high‐altitude areas, including data from questionnaires completed by 4988 Lhasa residents, and offers an updated overview of the burden these skin disorders impose on the general population. UV radiation, a major trigger for these conditions, poses a significant risk due to Lhasa's unique geographical location, high altitude, and intense UV exposure. Our findings reveal that the prevalence in Lhasa is 14.94% for melasma, 17.14% for plateau facial telangiectasia, and 0.38% for vitiligo. In contrast, previous research in plain areas indicated a melasma prevalence of 3.52% in Zibo [17], a facial telangiectasia prevalence of 10% in Queensland [4], and a vitiligo prevalence of 0.093% in Shaanxi Province [18]. These data underscore the heightened risk of melasma, facial telangiectasia, and vitiligo in high‐altitude areas.

Additionally, our study found that individuals aged 31–50 years show an increased prevalence of melasma and plateau facial telangiectasia. Across all demographics, women exhibited higher prevalence than men, and urban residents showed higher rates than their rural counterparts. Notably, women in their peak childbearing years (31–40) are particularly susceptible to melasma, while individuals aged 41–50 face a significant risk of plateau facial telangiectasia due to accumulated UV radiation damage. Moreover, a high prevalence of melasma was often accompanied by a high prevalence of plateau facial telangiectasia, indicating a strong correlation between these two conditions across different age groups, occupations, and genders. Finally, given the intense UV radiation characteristic of high‐altitude areas and the general lack of awareness regarding protective measures among the population, the screening and prevention of pigmented skin disorders remain crucial public health priorities that warrant further attention.

Previous studies had shown variations in the prevalence of pigmented skin disorders in different regions of the world, with a prevalence of melasma of 13.14%–15.5% in 401 Arab‐Americans [19] in 2007 and a prevalence of facial telangiectasia of 10% in 1400 Queensland community individuals [4] in 2011. The prevalence of vitiligo was 1.3% in 2022 among 35 694 participants from Europe, Japan, and the United States [20]. Differences in altitude, population characteristics, survey methods, socio‐economic levels, and public health construction levels may lead to inconsistent results, suggesting that the factors affecting the prevalence of pigmented skin disorder were complex. Our study found that the prevalence of pigmented skin disorder was related to gender, age groups, ethnic groups, and occupations, and more attention needs to be paid to women aged 31–50 years with long‐term exposure to high UV radiation.

Our study indicated that the prevalence of pigmented skin disorder was significantly higher in the high‐altitude area than in the plain, and the following potential reasons may explain this difference. Firstly, UV radiation is the main factor that induces or promotes the aggravation of melasma [21], People susceptible to sunburn easily have larger areas of facial telangiectasia than people who are not susceptible to sunburn, and long‐term exposure to sunlight can worsen facial telangiectasia [22, 23]. Melanocytes from vitiligo patients are also more susceptible to oxidative stress with UVB light in vitro [24], and women who sunburn easily 2 h after UV exposure and those with blistering sunburn are at higher risk for vitiligo [25]. These skin disorders are significantly related to UV radiation.

Our screening method demonstrated high sensitivity and specificity, affirming that our screening criteria are robust. Accompanying pictures enhances understanding, allowing primary healthcare workers to easily comprehend and effectively guide the screening process. This approach offers a simple and practical method for screening pigmented disorders in socially and economically disadvantaged areas.

However, this study has several limitations. Firstly, it was confined to a situational survey in Lhasa, without extending to other high‐altitude areas, which limits its generalizability. Secondly, it did not systematically explore potential influencing factors on skin disorders, such as diet, sleep patterns, smoking, other lifestyle habits, family history, and genetic predispositions. Thirdly, the reliance on self‐reported questionnaires might have introduced unavoidable errors, potentially biasing prevalence estimates. Furthermore, while efforts were made to correct the final sample, population migration, and exclusion criteria could have led to selection bias. Additionally, the screening criteria might have been subject to interpretation bias during investigator training, leading some patients and caregivers to inaccurately report skin conditions, thus excluding truly positive cases and skewing prevalence. Lastly, although the sample was from a high‐altitude area, it might not be fully representative, especially for patients residing in urban areas or regions with better healthcare access. Despite these limitations, our research still offers significant insights into the prevention and trends of pigmented skin disorders in high‐altitude areas, suggesting a user‐friendly screening tool. We hope our findings will contribute to the prevention and mitigation of pigmented skin disorders in China and other low‐ to middle‐income countries and regions.

In conclusion, our study revealed that the prevalence of three common pigmented skin disorders in Lhasa is higher than in some plain areas, with middle age and older age, outdoor work, and female gender identified as major risk factors. These findings highlight the critical situation of pigmented skin disorder prevention and control in high‐altitude areas, emphasizing the need for enhanced screening, education, and public health investment in prevention and treatment. Addressing these urgent issues is key to alleviating the burden of pigmented skin disorders in high‐altitude areas. Additionally, our study can serve as a foundation for screening similar skin conditions in other primary care settings and offer a feasible epidemiological research method for underprivileged areas.

## Author Contributions

Mingming Xu, Zha Zhen, Ying Chen and Wei Zhang had full access to all of the data in the study and takes responsibility for the integrity of the data and the accuracy of the data analysis. Concept and design: Zha Zhen, Ying Chen and Wei Zhang. Acquisition, analysis, or interpretation of data: Mingming Xu, Zha Zhen, Ying Chen, Shuo Zhang, Jing Li, Nan Li and Wei Zhang. Writing – original draft: Zha Zhen, Mingming Xu and Wei Zhang. Writing – review and editing: Mingming Xu, Wei Zhang. Statistical analysis: Ying Chen, Shuo Zhang, Jing Li, Nan Li and Ruiyu Li. Obtained funding: Wei Zhang and Ying Chen. Administrative, technical, or material support: Suolang Quzong, Zha Zhen, Liming Huang, Ying Chen and Wei Zhang. Supervision: Wei Zhang, Liming Huang, Ying Chen and Suolang Quzong.

## Ethics Statement

The study was approved by Ethics Committee of the Xizang Autonomous Region People's Hospital.

## Conflicts of Interest

The authors declare no conflicts of interest.

## Data Availability

The data that support the findings of this study are available from the corresponding author upon reasonable request.
